# Thoracic manifestations of IgG4‐related disease

**DOI:** 10.1111/resp.14422

**Published:** 2022-11-27

**Authors:** Romain Muller, Mikael Ebbo, Paul Habert, Laurent Daniel, Antoine Briantais, Pascal Chanez, Jean Yves Gaubert, Nicolas Schleinitz

**Affiliations:** ^1^ Internal Medicine Department, Hopital La Timone, APHM Aix Marseille University Marseille France; ^2^ Imaging Department, Hopital Nord, APHM Aix Marseille University Marseille France; ^3^ LIIE (Experimental Interventional Imaging Laboratory) Aix Marseille University Marseille France; ^4^ Anatomopathology Department, APHM Aix Marseille University Marseille France; ^5^ Pneumology Department, Hopital Nord, APHM Aix Marseille University Marseille France; ^6^ Imaging Department, Hopital La Timone, APHM Aix Marseille University Marseille France

**Keywords:** IgG4, immunoglobulin G4‐related disease, interstitial lung disease, rare systemic fibroinflammatory disease, thoracic

## Abstract

Immunoglobulin G4‐related disease (IgG4‐RD) is a recently described rare systemic fibroinflammatory disease with an estimated incidence of less than 1 in 100,000 persons per year. The disease can affect virtually any organ and is characterized by unifying histopathological findings. Recently, four subgroups of patients have been characterized: hepatobiliary, head and neck, Mikulicz syndrome and retroperitoneal fibrosis, who illustrate the mainly abdominal and ENT tropism of the disease. Yet, thoracic involvement is not uncommon. It can be detected in up to 30% of patients with systemic IgG4‐RD and is the exclusive manifestation of the disease in about 10% of cases. Clinical symptoms are nonspecific and may include dyspnoea, cough or chest pain. Chest CT findings are heterogeneous and primarily include peribronchovascular thickening, nodules, ground‐glass opacities and lymphadenopathy. There is no specific diagnostic test for IgG4‐RD thoracic involvement, which may mimic malignancy or vasculitis. Therefore, a cautious approach is needed to make an accurate diagnosis: a search for extra‐thoracic manifestations, elevated serum IgG4 levels, circulating levels of plasmablasts and pathologic evidence of disease is warranted. Although very suggestive, neither the presence of a polyclonal IgG4 lymphoplasmacytic infiltrate, storiform fibrosis or obliterative phlebitis are sufficient to confirm the histological diagnosis. Steroids are recommended as first‐line therapy. Rituximab or disease‐modifying antirheumatic drugs may be used in relapsed or rare cases of steroid‐refractory disease. In this review, we summarize current knowledge regarding the pathophysiology, epidemiology, diagnostic modalities (clinical–biological–imaging–histopathology) and treatment of IgG4‐RD thoracic involvement.

## INTRODUCTION AND HISTORY

Immunoglobulin G4‐related disease (IgG4‐RD) is a recently described systemic fibroinflammatory disease characterized by unifying histopathological findings.^1^ The disease can affect virtually any organ, either singly or in association, but most commonly involves lymph nodes, liver, pancreas, lacrimal and salivary glands.^2^

The description of the first cases of IgG4‐RD dates from 1961: Sarles et al. reported chronic autoimmune pancreatitis in patients with a distinctive histological feature of fibrosis and lymphoplasmacytic infiltration.^3^ In 2001, Hamano et al. reported fast‐moving band in the beta‐gamma region of the serum protein electrophoresis of patients with sclerosing pancreatitis, representing elevated IgG4 levels.^4^ In 2003, abundant polyclonal IgG4+ plasma cells were found within polyclonal lymphoplasmacytic infiltrates in tissue samples from patients with autoimmune pancreatitis, both in the pancreas and in the liver and gallbladder.^5^ Based on these findings, in 2006, Kamisawa proposed the existence of an IgG4‐related sclerosing disease corresponding to pancreatic and extrapancreatic fibroinflammatory lesions rich in IgG4.^6^ The disease has been variously named, including IgG4‐related autoimmune disease, IgG4‐associated multifocal systemic fibrosis, IgG4‐related systemic disease, IgG4‐related sclerosing disease or IgG4 plasmocytic syndrome. In 2011, an international group of investigators agreed on a uniform nomenclature and diagnostic criteria for this entity consensually referred to as *IgG4‐related disease*.^7^ This term emphasized the systemic infiltration of IgG4+ plasma cells in the organs involved and the frequency of high serum IgG4 levels without implying any pathogenic role of IgG4. This new nosological entity included many syndromes previously considered independent and idiopathic (inflammatory pseudotumours, Mikulicz syndrome, Küttner tumour, Riedel thyroiditis, mediastinal and retroperitoneal fibrosis).

Today, the use of the term IgG4‐RD has reached a consensus and is based on precise classification criteria published in 2019.^8^ It is a polymorphic systemic disease, whose most common presentations are hypertrophies of the salivary and lacrimal glands, lymphadenopathy, pancreatitis, sclerosing cholangitis, retroperitoneal fibrosis and tubulointerstitial nephritis.^9^ This wide heterogeneity has recently led to the description of four subgroups of patients (hepatobiliary, disease limited to the head and neck, Mikulicz and retroperitoneal fibrosis), whose pathophysiological, prognostic and therapeutic response particularities remain to be evaluated,^10^ but highlight the mainly abdominal and ENT tropism of the disease.

As a matter, the thoracic involvement of IgG4‐RD has been only recently described.^11^ The disease can affect nearly all thoracic organs, including heart, vessels, lungs, pleurae, lymph nodes, retromediastinum and bones. This thoracic heterogeneity has long been poorly characterized^12^ but is the subject of a growing number of publications. Here we review the current knowledge about pathogenesis, epidemiology, diagnostic strategies and therapeutic modalities of thoracic IgG4‐RD.

## PATHOGENESIS

### The pathogenesis of IgG4‐RD remains poorly understood

The presence of elevated serum IgG4 levels, the heavy infiltration of lesions by IgG4 plasma cells,^7^ the presence of high levels of circulating plamablasts,^13^ and the response to treatment with the B‐cell depleting agent Rituximab suggest hyperactivation of IgG4+ B cells in the disease.

IgG4 is the smallest subclass of immunoglobulin and represents about 5% of total IgG. It is a particular immunoglobulin that is usually considered non‐inflammatory because of its inability to activate the complement pathway^14^ and to generate immune complexes^15^ and its poor affinity for FcyR, leading to peak antibody‐dependent cell‐mediated cytotoxicity (ADCC).^16^ Consequently, the pathogenicity of IgG4 in IgG4‐RD is still not established. Patients with other diseases with markedly elevated serum IgG4 levels, such as IgG4 myeloma, do not develop IgG4 disease characteristics whereas about one‐third of patients with IgG4‐RD have normal IgG4 levels.^17^ Therefore, IgG4 could be a marker of the disease rather than a pathological factor: circulating B cells could differentiate into IgG4 B cells in tissues due to local tolerogenic environment, the elevated serum IgG4 level then being a consequence of the disease and not its source.^18^

Among the B cells involved in IgG4‐RD, plasmablasts (defined as CD19low CD38+CD20−CD27+ cells) have been the most studied.^19^ They are detected in organs involved in IgG4‐RD with activated phenotype (high expression of HLA‐DR, CD95, CD86 and CD62L) and evidence of active IgG4 secretion. They are also detectable in blood, where circulating levels correlate with disease activity and significantly decline after Rituximab‐induced remission.^20^ However, plasmablasts do not express CD20, the target of Rituximab, suggesting that the effect of Rituximab may be mediated by other B cells, which play a critical role upstream of plasmablasts activation. These may be memory B cells (CD19+CD20+CD27+CD38−), whose circulating levels have also been found to correlate with disease activity.^21^

Oligoclonal expansion of IgG4+ plasmablasts that occurs in IgG4‐RD patients is characterized by somatic hypermutations of the rearranged immunoglobulin heavy chains, suggesting T‐cell dependent stimulation. Among T cells, circulating follicular helper cells (Tfh) could play a major role in IgG4‐RD. Circulating Tfh cells produce IL‐4, which is involved in class‐switching of B cells to both IgG4 and IgE. Numbers of circulating Tfh cells correlate with number of circulating plasmablasts and serum IgG4 concentrations.^22^ More precisely, Tfh increase is characterized by specific expansion of Tfh2 (CCR6−CXCR3−) cells and to a lesser extent of T_FH_17 (CCR6+CXCR3−).^23^ Tfh2 cells are able to induce in vitro differentiation of naïve B cells into plasmablasts and to increase production of IgG4.^24^ IL‐10 is another cytokine involved in class‐switching from IgM to IgG4, which may be secreted by follicular T regulatory cells (Tfr).^25^ IL‐10‐producing Tfr are identified in both blood and tissues affected by IgG4‐RD.^26^ Circulating levels correlate with serum IgG4 concentrations, and the number of organs involved.

The existence of a type 2 helper (Th2) immune response in IgG4‐RD patients is supported by the increased expression of IL‐4, IL‐5 and IL‐13.^20^ Th2 response could be promoted by repeated exposure to an antigen (annexin A11 and galactin‐3 have both been recently implicated^27^), which is at the origin of the theory of an allergic mechanism, supported by the existence of an atopic background in patients, as well as the frequent presence of eosinophilia and elevated IgE.^28^ The role of this Th2 immune response is questioned.^29^ Population gene level analysis recently revealed a dominant gene signature for CD4+ cytotoxic T lymphocytes (CT) rather than for TH2 cells in IgG4‐RD. Circulating CD4+ SLAMF7+ CTL could play an important role in the pathogenesis of the disease.^30^ These cells are among the most abundant in involved tissues, close to IgG4 plasma cells and their number correlates with the extent of organ involvement.^30^ Finally, Treg are similarly increased in plasma and in sites affected by IgG4‐RD.^31^ Tregs secrete IL‐10 INFy and TGFb which can increase IgG4 production at the expense of IgG1 or IgE by B cells and promote the development of fibrosing lesions.^32^

Innate immunity is involved in the pathogenesis of IgG4‐RD. Anti‐inflammatory polarized M2 macrophages are implicated in tissue fibrosis in IgG4‐RD. These cells are observed in fibrotic areas and their number is correlated with the degree of tissue fibrosis.^33^ They could also promote the survival of plasma cells, which contribute to fibrosis through the production of PDGF‐B.^34^

Except for one case report of identical twins with IgG4‐RD,^35^ arguments for genetic susceptibility are limited. Paediatric onset of the disease is rare, but a review published in 2016 identified 25 cases in children,^36^ and another published in 2022 reported about 100 cases under 25 years of age were reported.^37^ A few cohort studies have identified genetic characteristics associated with IgG4‐RD. Some HLA haplotypes and CTLA4 alleles seem to be more frequent in IgG4‐RD patients,^38^ but the precise contribution of these genes to the pathophysiology of the disease remains to be determined.

## EPIDEMIOLOGY

To date, the incidence of the disease has been estimated in Japan to be approximately 1 per 100,000^39^ and prevalence has been estimated to be 0.8/100,000.^40^ The disease occurs predominantly in adults, with a median age at diagnosis of 60 years, and affects mainly men (1 man for 0.77 women). The age of onset, usually after 50 years, and the male predilection are unusual epidemiological parameters for an autoimmune disease.

Like IgG4‐RD, thoracic involvement is typically seen in middle‐aged to elderly adults, more commonly in men than women.^41^ It is estimated that 15 and 35% of patients with IgG4‐RD are affected.^10^, ^42^, ^43^, ^44^ Most cases of thoracic involvement with IgG4‐RD are found incidentally during the workup of extrathoracic lesions.^42^ The most common thoracic disorders seem to be bronchopulmonary disease and lymph node involvement.^44^

Isolated thoracic involvement was estimated to occur in 13% of patients.^42^, ^44^ Isolated lung involvement was estimated at 8% in a Japanese study based on 4304 patients.^39^

## CLINICAL PRESENTATION

IgG4‐RD usually manifests subacutely. More than half of the patients are asymptomatic according to several studies.^11^, ^42^, ^44^ Two small studies totaling 53 patients with systemic IgG4‐RD have detailed the general clinical signs present at diagnosis.^45^, ^46^ The main general symptoms included asthenia in 14 patients (25%), weight loss in 11 (20%) and fever in 4 (8%). Clinical signs of thoracic involvement could be present in more than 10% of the cases. They are nonspecific and depend on the location of the lesion, including cough, dyspnoea, haemoptysis, asthma or chest pain.^47^

## IMAGING PRESENTATION

Chest CT‐scan is the most widely performed imaging method to evaluate IgG4‐RD thoracic involvement. Chest CT‐scan presentation of IgG4‐RD is polymorphic and non‐specific.^11^, ^42^, ^48^, ^49^ Ideally, patients should be evaluated with dedicated thoracic imaging, including thin‐section CT scans and high‐resolution reconstruction filters. Chest CT scans should then be analysed by radiologists specialized in thoracic imaging and familiar with the disease. In our experience, specialized review in a tertiary centre significantly improved the number of abnormalities diagnosed. This was particularly pronounced in the case of pulmonary involvement, especially for discrete images of round shaped ground‐glass opacities (GGO) or fine reticulations.^44^

**FIGURE 1:**
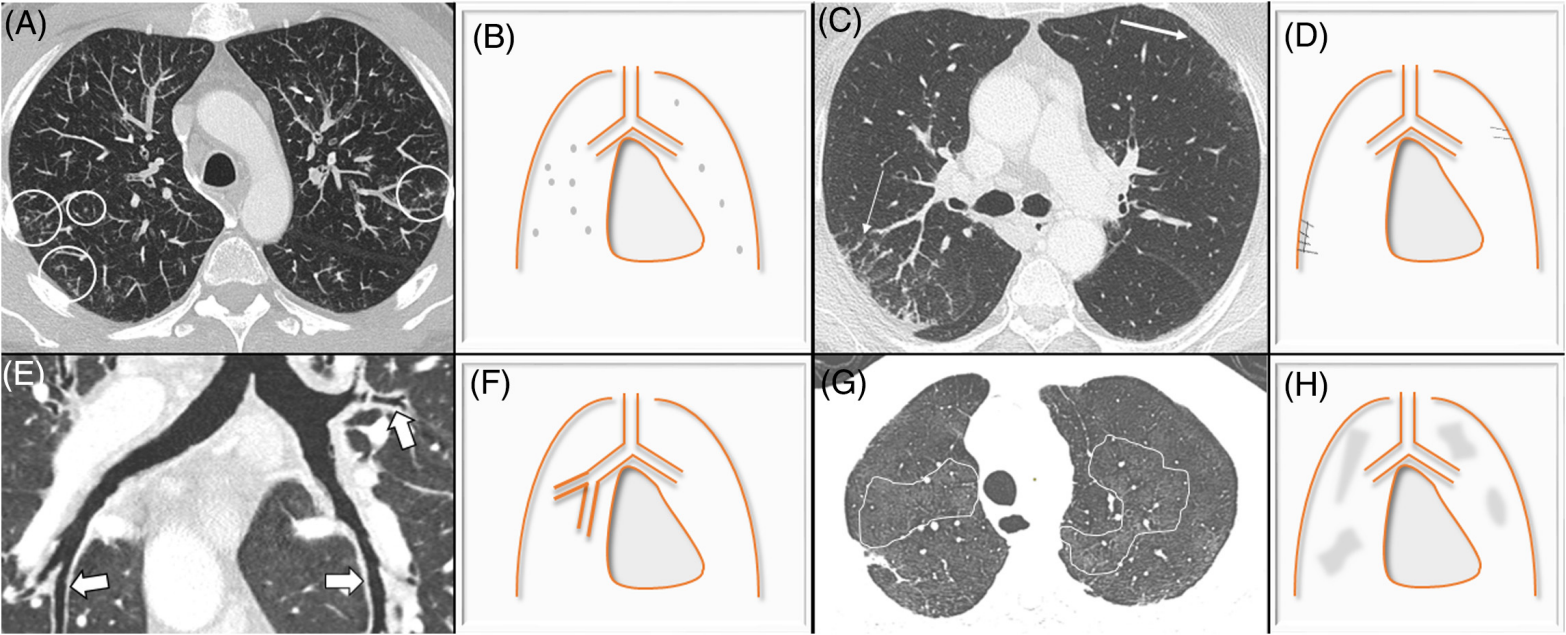
The four CT‐scan lung patterns of IgG4‐RD involvement. Axial chest CTs in parenchyma window associated with illustrations of the four lungs patterns. (1) *Nodular pattern*: small or pericentimers nodules randomly dispose (inside the circles). A 4 mm thickness with maximal intensity projection was performed to highlight the abnormalities (A,B). (2) *Interstitial disease pattern*: fine bilateral subpleural lines and not only in the posterior localization (thin arrows) (C,D). (3) *Peribronchovascular pattern*: diffuse bronchial wall thickening in curvilinear reconstruction (white arrows) (E,F). (4) *Ground‐glass opacities (GGO) pattern*: area of GGO highlighted with 4 mm thickness minimal intensify projection reconstruction and circled (G,H).

**FIGURE 2:**
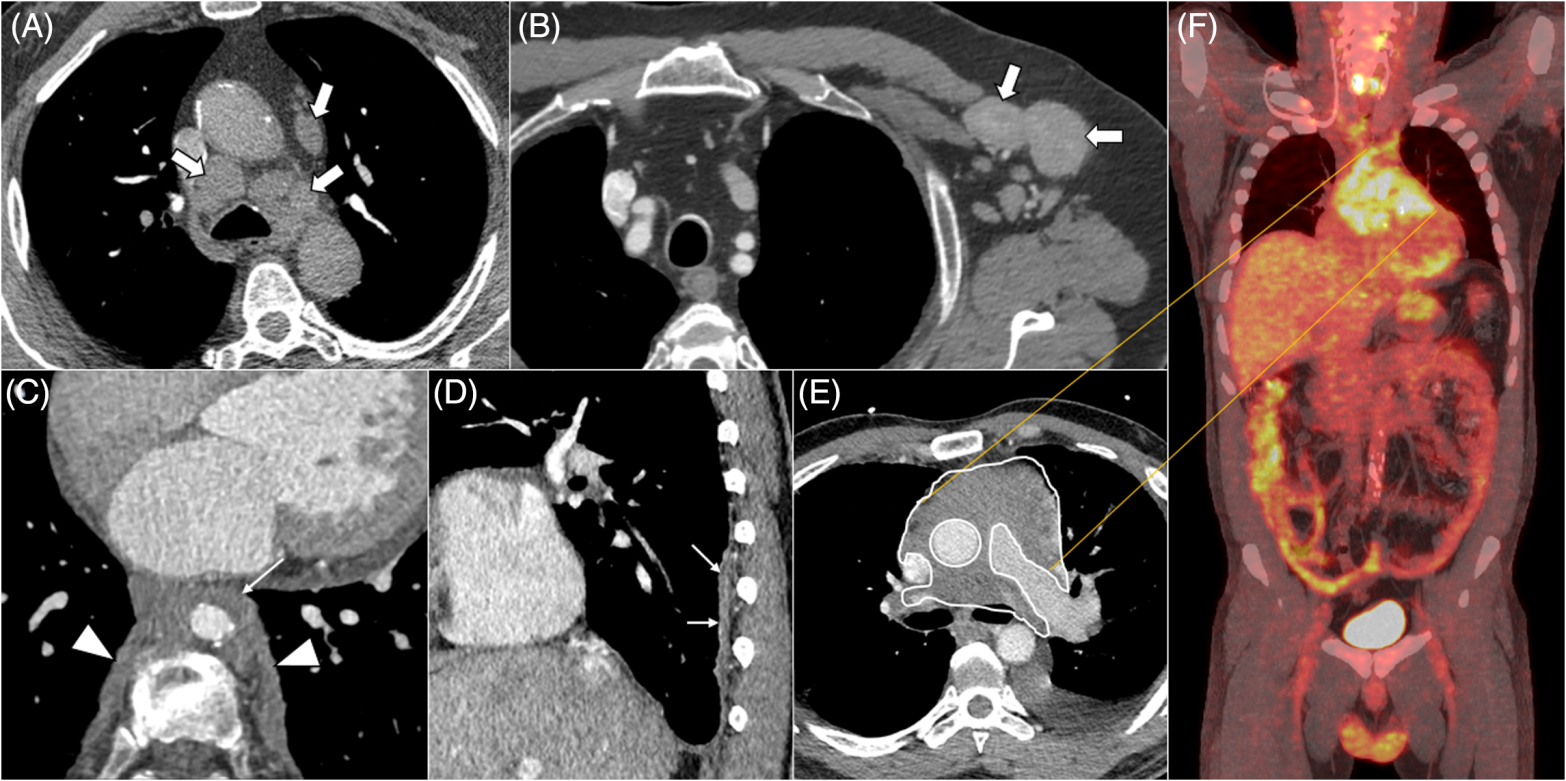
Examples of thoracic extra pulmonary features of IgG4‐RD involvement. Examples of enhanced chest CT scans in mediastinum windows and PET‐CT illustrating thoracic extra pulmonary involvements of IgG4‐RD: (1) *Lymph node pattern*: enlarged lymph nodes in the mediastinum (white arrows) with homogenous contrast uptake (A) and Enlarged lymph nodes in the left axilla (white arrows) showing homogenous contrast uptake and smooth borders (B). (2) *Retromediastinal pattern*: Typical bilateral paravertebral soft bands (white arrowheads), associated with a circumferential aortitis and responsible for an aortic stenosis (thin white arrow) (C). (3) *Pleural pattern*: Right side pleural thickening (thin white arrows) (D). (4) *An example of a rare off‐pattern condition*: anterior mediastinitis (circled area) with 18FDG uptake on coronal merged of CT and PET (E,F).

Through literature, seven main patterns of thoracic IgG4‐RD involvement are described^11^ and can be associated. Four patterns affect lungs: nodular, GGO, interstitial disease and peribronchovascular (Figure 1). The three others correspond to chest extrapulmonary lesions: lymph node, pleural and retromediastinal patterns (Figure 2). In the framework of the latter, the presence of a paravertebral soft band is relatively specific of the disease. This corresponds to a thickening of the paravertebral sulcus, in the lower thoracic region involving two or more vertebrae in a row. This sign is mostly located on the right side (97%, *n* = 31).^50^ Other CT‐scan findings than those included in these seven patterns are possible, but much rarer.

The relative frequency of these different patterns fluctuates between studies. In a cohort of 48 patients with thoracic involvement, we found that peribronchovascular involvement was the most frequent, affecting 60% of those patients. Fujinaga et al.^51^ identified hilar lymph node involvement as the most prevalent (80%, *n* = 90). The GGO pattern was the most common according to Inoue et al.^11^ (70%, *n* = 13), and Seraya et al.^43^ (70%, *n* = 16), whereas it was the nodular pattern in the study by Kang et al. (30%, *n* = 37).^52^ None of these patterns is specific for IgG4‐RD. These discrepancies could be partly explained by different patient recruitment methods, bias related to retrospective data collection, the lack of central imagery reviewing and different pattern definitions from one study to another.

Even in the setting of well‐known IgG4‐RD, any chest imaging abnormalities are not necessarily secondary to IgG4‐RD. Thus, classification criteria for thoracic involvement have been developed by Corcoran et al. to assess the responsibility of the disease in observed thoracic lesions.^42^ According to their criteria, thoracic involvement is categorized between ‘definite’ (biopsy‐proved), ‘highly probable’ (typical imaging finding with no alternative explanation and response to treatment), ‘probable’ (typical radiology with no alternative explanation) and ‘possible’ (radiology consistent but plausible alternative cause identified). In the absence of biopsy or evaluation of the effect of a test treatment, the association of two or more thoracic patterns in the same patient could suggest IgG4‐RD.

Some thoracic imaging patterns might be associated with specific IgG4‐RD extra‐thoracic involvements. For example, our previous study of 48 patients with IgG4‐RD thoracic involvement suggested that round‐shaped GGO may be associated with a more frequent IgG4‐related pancreatitis, that lymph node enlargement might be associated with a greater frequency of sialadenitis, and that interstitial disease could be associated with a more frequent hypereosinophilia.^44^ Underlying pathophysiological mechanisms are not established, and these data need to be confirmed by further studies.

^18^F‐FDG PET/CT can also be used for a comprehensive assessment of organ involvement. It could have greater sensitivity for detecting disease in arteries (aortitis and periaortitis), salivary glands and lymph nodes.^53^ ^18^F‐FDG PET/CT can be useful detecting clinically asymptomatic IgG4‐RD organ involvement in the thorax, then selecting the most diagnostically informative biopsy site, assessing response to therapy and monitoring for disease relapse.^54^

## BIOLOGICAL PRESENTATION

Biologically, two key markers for diagnosis and follow‐up of the disease have been identified to date: serum IgG4 level and detection of circulating plasmablasts.

Elevated serum IgG4 concentration (>135 mg/dl) represents an important diagnostic consideration. This threshold is part of all classification and diagnosis criteria, including the last ACR‐EULAR criteria published in 2019, but exhibits inconstant sensibility and specificity and so a low positive predictive value (34%) for the diagnosis of IgG4‐RD.^55^ Overall, it is estimated that 60% of patients with IgG4‐RD have a serum IgG4 level greater than 135 mg/dl, but this percentage varies according to ethnicity and the number of organs affected. Caucasian patients cohorts report less than 50% elevation in IgG4,^56^ compared to over 90% in Asian cohorts,^57^ and patients with multiple organ damage have significantly higher IgG4 levels.^58^ Markedly elevated serum IgG4 is rather more specific for IgG4‐RD: a 5 g/L cut off is associated with a specificity of 90%.^59^ Of note, IgG subclasses are most often measured by immunonephelometry, a technique that may show limitations due to prozone effect and interactions of IgG4 with measurement of IgG1 and IgG2, which is why some authors have proposed the preferential use of mass spectrometry in IgG4‐RD.^60^

Flow cytometric detection of circulating plasmablasts is also a useful biomarker for diagnosing IgG4‐RD. A 900/ml cut‐off could be associated with reported sensitivity more than 90% and specificity of 80%.^13^ This biomarker was found to correlate with disease activity and the number of affected organs, independently of serum IgG4 levels.^61^ The levels of other circulating cells identified during IgG4‐RD (CD4+ CTL, Tfh, B memory cells) are correlated with disease activity but clear thresholds between healthy and diseased individuals remain to be determined.

Moreover, a nonspecific biological inflammatory syndrome is often noted. Elevated C‐reactive protein levels are found in a quarter of patients, but this increase is usually modest.^62^ Polyclonal hypergammaglobulinemia (total serum IgG levels >1800 mg/dl) is reported in 60% of the patients, which may be associated with high IgG2 and low IgA levels.^63^ Other non‐specific biologic markers observed include hypocomplementemia (40%), high soluble IL‐2R levels, positivity of antinuclear antibody (30%) or rheumatoid factor (20%), eosinophilia (30%) and elevated IgE (60%), especially in patients with asthma and atopy. The combination of asthma, hypereosinophilia and elevated IgE is not uncommon in IgG4‐RD and seems to be correlated with the risk of disease recurrence.^64^ However, at the time of diagnosis, this association should raise the possibility of atopic asthma, hypereosinophilic syndrome (HES), ANCA vasculitis or immunoallergic aspergillosis. For this reason, hypereosinophilia greater than 3G/L constitutes an exclusion factor for the disease according to the ACR/EULAR classification criteria.

Many other markers, that we not detailed in this review, are currently under investigation in IgG4‐RD.^63^ Summary of blood biomarkers used for diagnosis, assessment of disease activity and prediction of relapse risk is presented in Table 1.

**TABLE 1:** Summary of blood biomarkers used for diagnosis, assessment of disease activity and prediction of the risk of relapse

	Blood biomarker	Threshold
For diagnosis	Serum IgG4^65^	>135 mg/dl (sensitivity 80%, specificity 80%)
	Circulating plasmablasts^13^	>900/ml (sensitivity 90%, specificity 80%)
To assess disease activity	Serum IgG4^65^	>135 mg/dl
	Circulating plasmablasts^13^	>900/ml
	Serum IgE^64^	>125 UI/ml
	Serum IgG^64^	>1800 mg/dl
	ESR^66^	>20–25 mm/h
	CRP^66^	>5–10 mg/L
	C3^57^	<80 mg/dl
	C4^57^	<15 mg/dl
	soluble IL‐2R^67^	<2500 pg/ml
	Circulating CD4+ CTL^32^	Not defined to date
	Circulating Tfh^22^	Not defined to date
	Circulating B memory cells^21^	Not defined to date
To predict the risk of relapse	Serum IgG4^65^	>135 mg/dl^a^
	Circulating plasmablasts^13^	>900/ml^a^
	Serum IgE^64^	>125UI/ml^a^
	C3^57^	<80 mg/dl^a^
	C4^57^	<15 mg/dl^a^
	Circulating eosinophils^68^	>0.5 G/L^a^

^a^ Or increase from remission rate.

## HISTOPATHOLOGY OF THORACIC LESIONS

IgG4‐RD demonstrates common histologic features in most of organs that may be affected. The three main histopathological characteristics of IgG4‐RD are the presence of a polyclonal lymphoplasmacytic infiltrate, a storiform fibrosis and obliterating phlebitis.^2^

The dense lymphoplasmacytic infiltrate is composed of T cells, B cells, occasional germinal centres and plasma cells. It can cause obliteration of the venous channels, leading to obliterative phlebitis. An essential feature of this infiltrate is the abundant presence of IgG4 plasma cells, which should be assessed semi‐quantitatively by immunostaining of the total number of IgG4 cells per high‐power field (/hpf, whose positivity thresholds depend on the site of involvement) and measurement of the IgG4/IgG plasma cell ratio (generally considered abnormal if the ratio is >40%).^7^ Storiform fibrosis is a swirling, ‘cartwheel’ pattern of fibrosis sometimes with a patchy distribution that may be missed with small biopsies. Other ‘minor’ histopathological features include increased number of eosinophil and phlebitis without obliteration of the lumen. Absence of granulomatous inflammation and prominent neutrophilic infiltrate is required. Histological findings are neither specific nor sensitive and may lead to under or overdiagnosis.^69^ This resulted in a probabilistic histologic classification of the disease into three groups: ‘histologically highly suggestive of IgG4‐RD’ (presence of at least two of the three characteristic histological features), ‘probable histologic features of IgG4‐RD’ (only one of them) and ‘insufficient histopathologic evidence of IgG4‐RD’ (none of them), as it may be the case after previous therapy or progression to a fibrotic stage.^7^

At the thoracic level, different organs can be investigated, with few histological specificities. Regarding lymphadenopathies, five morphological subtypes have been described,^69^ but the specificity of these histologic changes in the absence of other evidence of IgG4‐RD remains controversial: (a) multi‐centric Castleman disease‐like, (b) reactive follicular hyperplasia, (c) interfollicular expansion, (d) progressive transformation of germinal centre‐like and (e) inflammatory pseudotumour‐like. The most specific subtype is the last mentioned, but reactive follicular hyperplasia is most frequently observed.^70^ Fibrosis is only seen in inflammatory pseudotumour‐like lesions, and obliterative phlebitis is rarely observed.^7^ IgG4+/plasma cells threshold is >50 in lymphadenopathies. Considering the variability of histologic patterns, the relative rarity of storiform fibrosis and obliterating phlebitis and the poor specificity of increased IgG4+ plasma cells in lymphadenopathies, the main value of lymph node biopsy is often to rule out differential diagnoses, such as lymphoma.

Regarding the lungs, histopathology of lesions shows diffuse lymphoplasmacytic infiltration, obliterative vascular changes and fibrosis with occasional eosinophilic infiltration.^71^ The most frequent histopathological pattern is a lymphangitic distribution of inflammatory infiltrates rich in plasma cells. Several specificities should be noted. Arteritis characterized by a lymphoplasmacytic infiltrate is regularly observed and may lead to obliteration.^72^ This obliterating arteritis is more frequent than obliterating phlebitis, unlike in other organs.^7^ In addition, the characteristic storiform fibrosis seen in other organs is rarely found into pulmonary lesions.^73^ Consensus thresholds are >50 IgG4/plasma cells (/hpf) for surgical biopsies but >20 IgG4/ plasma cells (/hpf) for nonsurgical biopsies.^7^ IgG4/IgG ratio threshold remains the same (>40%) for both types of biopsies.

Regarding the pleura, histological patterns correspond to those commonly observed in most of the other affected organs, with a threshold of >50 IgG4/plasma cells (/hpf).^74^ In the mediastinum, histopathology of lesions exhibits storiform cell‐rich fibrosis, lymphoplasmacytic infiltrate with IgG4‐positive plasma cells and obliterative phlebitis,^75^ which are not specific, and may be observed, for example, in histoplasmosis.^76^ Consensus statement on the pathology of IgG4‐related disease published in 2012 did not propose a specific IgG4/plasma cell threshold for fibrosis mediastinitis. The one suggested proposed for retroperitoneum (30IgG4/plasma cells) is therefore most often retained by analogy.

Several procedures are available to collect a sample of involved tissue, depending on the organ concerned, the location of the lesion, the patient's medical history and the experience of the centre. Mediastinal lymph nodes can be reached by endobronchial ultrasound (EBUS‐TBNA), surgical sampling or mediastinoscopy, while pleural involvement can be reached by percutaneous core cutting needle or surgical sampling.^44^ There are no studies comparing the cost‐effectiveness of these different techniques in the specific setting of IgG4‐RD. Nevertheless, given the relevance of lymphadenopathies architecture analysis in the diagnosis of IgG4‐RD, biopsy or surgical excision should be preferred to EBUS‐TBNA. If thoracic affected organs are not accessible for biopsy, a minor salivary gland biopsy may be performed, even without clinical signs of salivary involvement. Histologic specificities include possible conspicuous lymphoid follicle formation, and sometimes lacks obliterative phlebitis and storiform fibrosis. Moriyama et al. reported a sensitivity of 55% and a specificity of 100% for labial salivary gland biopsy involving 66 patients with suspected IgG4‐RD.^77^

## DIAGNOSTIC OF THORACIC IGG4‐RD

The diagnosis of thoracic involvement of IgG4RD is a challenging process based on the agreement of clinical, biological, imaging and histological findings. Virtually, all intrathoracic organs can be affected (Table 2) but no feature is pathognomonic for the diagnosis. The diagnostic approach must therefore always consider screening for differential diagnoses before retaining the diagnosis, especially when the disease affects a single organ and is either identified incidentally on radiological studies or diagnosed unexpectedly in pathological specimens.

**TABLE 2:** Thoracic manifestations of IgG4‐RD with their estimated frequency (in bold, the four most frequent organ involvement corresponding to the seven main thoracic patterns on CT scan)

Involved organ	Brief description of damages	Estimated frequency within thoracic involvement
**Lung**	Pulmonary infiltration and/or fibrosis that may be classified into four CT‐scan patterns: nodular, GGO, interstitial disease and peribronchovascular	40%–80%^42^, ^44^, ^48^, ^72^
**Lymphadenopathy**	Lymphadenopathy infiltration and/or fibrosis resulting in multiple lymphadenopathies in mediastinum and hilum	50%–80%^42^, ^44^, ^48^, ^72^
**Pleurae**	Pleural infiltration and/or fibrosis resulting in nodular or diffuse pleural thickening and/or pleural effusion	8%–15%^42^, ^44^, ^48^, ^72^
**Retromediastinum**	Retromediastinal infiltration and/or fibrosis, that may lead to vascular stenosis	3%–6%^42^, ^44^, ^48^, ^72^
Aorta	Thoracic aorta inflammation (vascular *wall contrast* enhancement), infiltration and/or fibrosis, associated with thickening of vascular walls that may lead to stenosis	1%^78^
Airway	Airway infiltration and/or fibrosis resulting in airway stenosis leading to atelectasia or asthma‐like symptoms	Less than 1%
Pulmonary vessels	Infiltration and/or fibrosis of pulmonary arteries that may lead to pulmonary hypertension	Exceptional
Heart	Coronary stenosis, valvular lesions, myocardial pseudotumour or pericardial effusion or thickening	Exceptional
Bones	Bone erosions adjacent to a pseudotumour	Exceptional

Clinically, the presence of extra‐thoracic organ involvement, especially in organs frequently affected by IgG4‐RD (pancreatitis, sclerosing cholangitis, lymphadenopathy, interstitial nephritis, sialadenitis and retroperitoneal fibrosis), supports the diagnosis. However, thoracic involvement may be isolated.^42^, ^44^ Increased serum IgG4 levels support the diagnosis, but may be normal in IgG4‐RD while elevated in other conditions such as recurrent infections, cancers or autoimmune diseases.^79^ Histological analysis of at least one affected organ is most often required for the diagnosis. But here again, histology is susceptible to abuse: some conditions such as Castleman's disease or Rosai–Dorfman's disease may be responsible for IgG4‐RD‐like features.^12^, ^47^ Furthermore, HES and eosinophilic granulomatosis and polyangiitis (mostly ANCA‐negative phenotype) may be responsible for genuine overlapping syndromes with IgG4‐RD, defining a spectrum of eosinophil‐mediated diseases that is still poorly characterized.^28^, ^80^, ^81^

**FIGURE 3:**
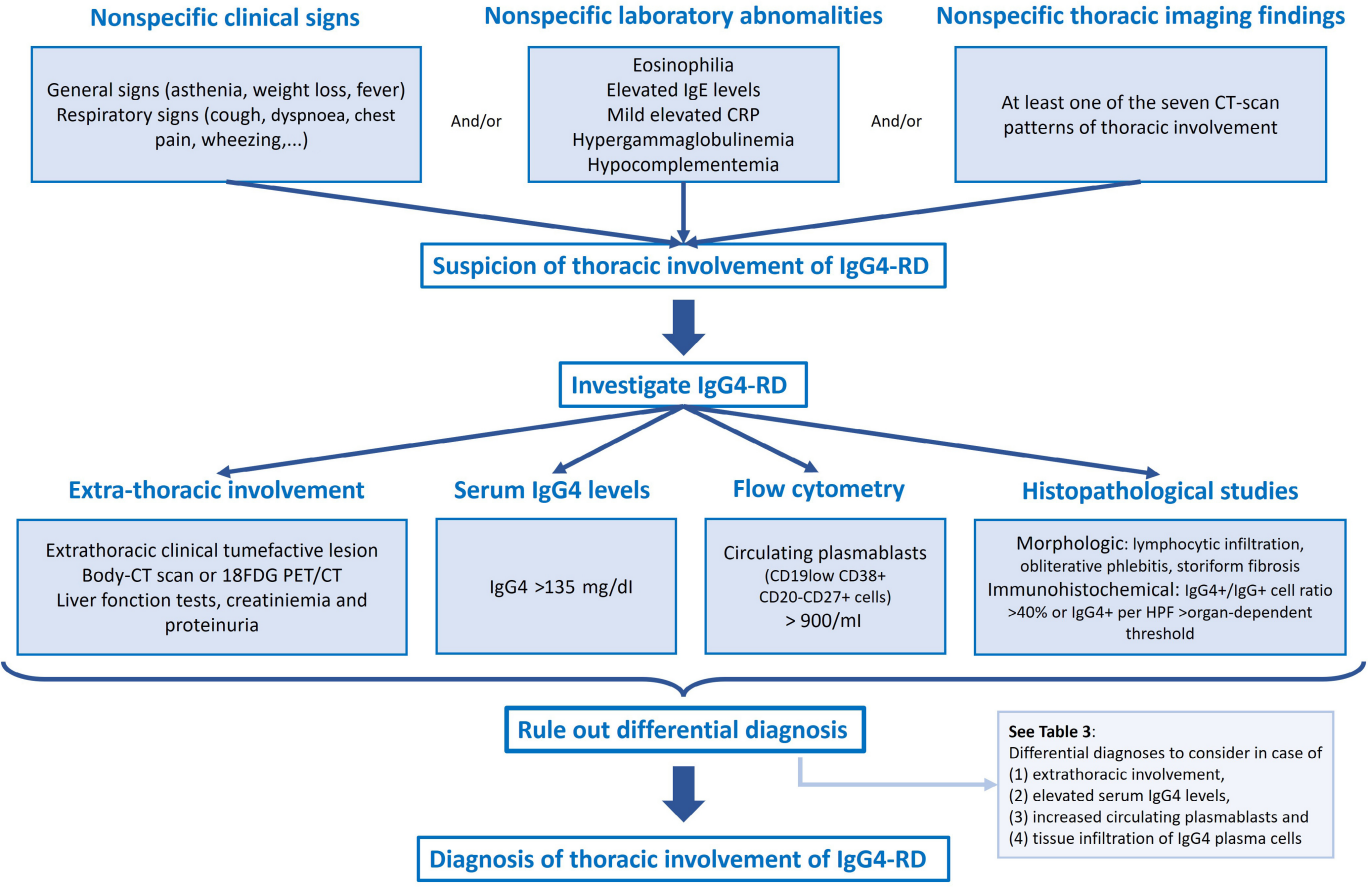
Diagnostic algorithm in thoracic IgG4‐RD.

The diagnostic approach (Figure 3) therefore consists of collecting all the clinical, biological, imaging and histological elements in favour of the diagnosis, while keeping in mind that each of these elements must raise specific differential diagnoses (Table 3). Pragmatically, patients who present with classic clinical, laboratory and radiological manifestations of IgG4‐RD but in whom biopsies are not feasible or noncontributory are generally given a working diagnosis of ‘suspected IgG4‐RD’ and treated as such, provided that sufficient efforts have been made to exclude IgG4‐RD mimics.^82^ Among IgG4‐RD mimetics, sarcoidosis (interstitial lung disease, adenopathy, granulomatous sialadenitis, increased serum CEA levels), ANCA vasculitis (interstitial lung disease, sinusitis, hypereosinophilia, glomerulonephritis, anti MPO or PR3 antibodies), neoplasia (altered general condition, adenopathy, pulmonary nodules or carcinomatous lymphangitis) and tuberculosis (pulmonary nodules, adenopathy and extra‐pulmonary involvement) must be considered systematically in case of pulmonary involvement.

**TABLE 3:** Differential diagnosis for IgG4‐related thoracic disease

*Diseases with similar thoracic disorder and extra‐thoracic involvement* ^47^ Connective tissue disease with thoracic involvement (e.g., Sjogren's syndrome…) Vasculitis with thoracic involvement (e.g., ANCA vasculitis…) Sarcoidosis, histiocytosis HES Metastatic neoplasia or lymphoma Infections with multisystem involvement (e.g., tuberculosis, histoplasmosis…)
*Diseases with similar thoracic disorder and increased serum IgG4 levels* ^79^ Repeated pulmonary infections Cystic fibrosis associated with colonization with Pseudomonas aeruginosa Connective tissue diseases, vasculitis, HES Neoplastic diseases
*Diseases with similar thoracic disorder and increased circulating plasmablasts levels* ^13^ ANCA vasculitis Sarcoidosis Sjogren's syndrome
*Diseases with similar thoracic disorder and tissue infiltration of IgG4 plasma cells* ^7^ ANCA vasculitis Multicentric Castleman's disease Histiocytosis (Rosai–Dorfman disease, Erdheim Chester disease) Infections (EBV, tuberculosis, histoplasmosis) Lymphoma and peritumoral infiltrate HES Cutaneous and systemic plasmocytosis, Lymphomatoid granulomatosis

## CLASSIFICATION AND STAGING

Classification and diagnosis criteria for IgG4‐RD have been published periodically since the recognition of the disease.

The most recent classifications are the ACR/EULAR criteria, published in 2019.^8^ They are based on a score in which clinical, biological, imaging and histological findings are considered. Thoracic involvement is included through ‘pulmonary involvement’, in which only the aspects of peribronchovascular and septal thickening are recognized (+4 points), and ‘retromediastinal involvement’ (paravertebral band‐like soft tissue in the thorax, +10 points). A score of 20 or more classifies patients as having IgG4‐RD.

In 2017, comprehensive diagnostic criteria were published by Umehara et al., revised in 2021.^83^ They are based on the combination of CT‐objectified thoracic organ involvement, IgG4 levels above 135 mg/dl, and IgG4+ >10/HPF cell infiltration associated with a ratio of IgG4+/IgG >40%. According to this work, the presence of these three parameters characterizes the ‘defined’ disorders.

In order to standardize the criteria for evaluating response to treatment, which may vary from one specialty to another, an IgG4‐RD Responder Index was developed in 2012.^84^ This score has been shown to have a good correlation with the physician global assessment.^85^ Disease activity in 14 organs is rated from 0 to 4. The degree of urgency of damages and sequelae are also recorded, and serum IgG4 level are included in the scoring. Within thoracic involvement, lung, lymphadenopathy, large vessel including aorta and mediastinum are especially mentioned. This score is now used in randomized clinical trials to evaluate response to treatments and its follow‐up over time.

## TREATMENT

Treatment of all symptomatic patients is recommended. Conversely, asymptomatic patients, such as those with isolated chest adenopathy, may remain untreated. Treatment of localized forms of the disease, such as a pulmonary nodule or inflammatory pseudotumour, may be limited to surgical removal. In other cases, the treatment of IgG4‐RD thoracic disease is based on immunosuppressive drugs.

International consensus statement on the treatment of IgG4‐RD has been published in 2015,^86^ based on expert opinion and review of available evidence. Steroids are suggested as a first line treatment, at 0.6 mg/kg. Efficacy is usually observed within the first 2 weeks of treatment.^87^ After 2–4 weeks of treatment at 0.6 mg/kg, steroids are usually tapered off over 3–6 months. Non‐response to steroids is uncommon (less than 5% of patients) and should lead to consideration of alternative diagnoses to IgG4‐RD. On the contrary, relapse following steroid reduction is quite common. It is estimated to occur in 60%–80% of patients.^88^ Steroid dependence may require the prescription of a second immunosuppressive drug to minimize the side effects of long‐term steroid use. It may also require the maintenance of very low doses of steroids over time (e.g., 5 mg/day), which decrease relapse rates compared to placebo.^89^

Rituximab is an anti‐CD20 monoclonal antibody that has been shown to be effective in reducing steroid dosage in IgG4‐RD patients with steroid dependence. The treatment is usually prescribed at the dose of 1 g on Day 1 and then on Day 15. Its effectiveness is generally maintained for several months. In the study by Carruther et al.,^90^ 97% of patients (29 out 30) experienced a favourable response to Rituximab, and 40% maintained complete remission at 1 year. Ebbo et al. reported 93.5% of clinical response (29 out 31 patients), with 42% of relapse at 2 years.^91^ Maintenance therapy at a dose of 1 g every 6 months is possible to limit the risk of late relapse or to control the onset of disease recurrence. Secondary hypogammaglobulinemia may then occur (3 patients out 31 Ebbo et al.), possibly associated with secondary infections, mainly affecting of the respiratory tract.

Disease‐modifying anti‐rheumatic drugs (azathioprine, mycophenolate mofetil, ciclosporin, methotrexate, …) are not very effective for induction of remission but may be used for steroids sparing.^86^, ^92^ Because of the lack of prospective clinical trials, the international consensus guideline on the treatment of IgG4‐RD had only 50% agreement among experts on whether disease‐modifying antirheumatic drugs should be started early or not.^86^

Several emerging therapies offer promise. A phase 2 clinical trial of anti‐CD19 antibody inebilizumab showed an interesting outcome in 12 patients (decrease in IgG‐RD Responder Index score by ≥2 points).^93^ The use of lymphoma chemoimmunotherapy regimens such as fludarabine or bendamustine in combination with rituximab has been successfully reported for steroid and rituximab‐refractory cases.^59^ The use of dupilumab, an anti‐IL4/IL‐13 monoclonal antibody prescribed to treat asthma, seems interesting given the involvement of these two cytokines in the pathophysiology of the disease, but its efficacy has only been reported in isolated cases and remains controversial.^94^, ^95^ Abatacept, a CTLA4‐Fc fusion protein, has also been evaluated in a few case report and a proof of concept study.^96^, ^97^ Studies are ongoing to evaluate the effectiveness of rilzabrutinib (BTK inhibitor) and elotozumab (anti‐SLAM7). Anti‐CD38 (daratumumab) or proteasome inhibitors (bortezomib) also offer interesting perspectives to specifically target B cells. Anti‐IgE (omalizumab) or anti‐IL5 (mepolizumab) treatment may be options for patients with atopy or severe asthma with high IgE levels.

## CONCLUSION

IgG4‐RD is a fibroinflammatory disease that can affect virtually all organs, mainly occurring in middle‐aged to elderly male patients. In the thorax, the disease can occur in all compartments (mediastinum, airways, lungs and pleurae), most often in association with extra‐thoracic manifestations. Clinical manifestations are nonspecific and usually lacking. CT‐scan findings can be divided into seven distinctive patterns, whose prognostic characteristics have yet to be determined. Diagnosis is based on a combination of clinical, biological and histological findings, as no marker is specific for the disease. Diagnostic approach must always consider screening for differential diagnoses especially when the disease affects a single organ, is identified incidentally on radiological studies, or is diagnosed unexpectedly in pathological specimens. Thoracic IgG4‐RD is generally highly responsive to immunosuppressive therapy with steroids and/or rituximab, but relapse is common. Further studies are needed to better characterize thoracic involvement of the disease, its triggers, pathophysiology and prognostic factors to elucidate more effective and specific therapies.

## AUTHOR CONTRIBUTION

Romain Muller, Mikael Ebbo and Nicolas Schleinitz conceived and designed the work. Romain Muller and Mikael Ebbo drafted the article. Paul Habert, Laurent Daniel, Antoine Briantais, Pascal Chanez, Jean Yves Gaubert and Nicolas Schleinitz critically reviewed the manuscript.

## CONFLICT OF INTEREST

None declared.

## Data Availability

The data that support this manuscript are available from the corresponding author, (Nicolas Schleinitz), upon request.

## References

[1] Stone JH , Khosroshahi A , Deshpande V , Chan JKC , Heathcote JG , Aalberse R , et al. Recommendations for the nomenclature of IgG4‐related disease and its individual organ system manifestations. Arthritis Rheum. 2012;64:3061–7. 22736240 10.1002/art.34593 PMC5963880

[2] Stone JH , Zen Y , Deshpande V . IgG4‐related disease. N Engl J Med. 2012;366:539–51. 22316447 10.1056/NEJMra1104650

[3] Sarles H , Sarles JC , Muratore R , Guien C . Chronic inflammatory sclerosis of the pancreas—an autonomous pancreatic disease? Am J Dig Dis. 1961;6:688–98. 13746542 10.1007/BF02232341

[4] Hamano H , Kawa S , Horiuchi A , Unno H , Furuya N , Akamatsu T , et al. High serum IgG4 concentrations in patients with sclerosing pancreatitis. N Engl J Med. 2001;344:732–8. 11236777 10.1056/NEJM200103083441005

[5] Kamisawa T , Funata N , Hayashi Y , Eishi Y , Koike M , Tsuruta K , et al. A new clinicopathological entity of IgG4‐related autoimmune disease. J Gastroenterol. 2003;38:982–4. 14614606 10.1007/s00535-003-1175-y

[6] Kamisawa T , Okamoto A . Autoimmune pancreatitis: proposal of IgG4‐related sclerosing disease. J Gastroenterol. 2006;41:613–25. 16932997 10.1007/s00535-006-1862-6 PMC2780632

[7] Deshpande V , Zen Y , Chan JK , Yi EE , Sato Y , Yoshino T , et al. Consensus statement on the pathology of IgG4‐related disease. Mod Pathol. 2012;25:1181–92. 22596100 10.1038/modpathol.2012.72

[8] Wallace ZS , Naden RP , Chari S , Choi H , Della‐Torre E , Dicaire J‐F , et al. The 2019 American College of Rheumatology/European league against rheumatism classification criteria for IgG4‐related disease. Arthritis Rheumatol. 2020;72:7–19. 31793250 10.1002/art.41120

[9] Mahajan VS , Mattoo H , Deshpande V , Pillai SS , Stone JH . IgG4‐related disease. Annu Rev Pathol. 2014;9:315–47. 24111912 10.1146/annurev-pathol-012513-104708

[10] Wallace ZS , Zhang Y , Perugino CA , Naden R , Choi HK , Stone JH , et al. Clinical phenotypes of IgG4‐related disease: an analysis of two international cross‐sectional cohorts. Ann Rheum Dis. 2019;78:406–12. 30612117 10.1136/annrheumdis-2018-214603 PMC6996288

[11] Inoue D , Zen Y , Abo H , Gabata T , Demachi H , Kobayashi T , et al. Immunoglobulin G4‐related lung disease: CT findings with pathologic correlations. Radiology. 2009;251:260–70. 19221056 10.1148/radiol.2511080965

[12] Campbell SN , Rubio E , Loschner AL . Clinical review of pulmonary manifestations of IgG4‐related disease. Ann Am Thorac Soc. 2014;11:1466–75. 25422997 10.1513/AnnalsATS.201403-128FR

[13] Wallace ZS , Mattoo H , Carruthers M , Mahajan VS , Della Torre E , Lee H , et al. Plasmablasts as a biomarker for IgG4‐related disease, independent of serum IgG4 concentrations. Ann Rheum Dis. 2015;74:190–5. 24817416 10.1136/annrheumdis-2014-205233 PMC4656194

[14] Peng L , Lu H , Zhou J , Zhang P , Li J , Liu Z , et al. Clinical characteristics and outcome of IgG4‐related disease with hypocomplementemia: a prospective cohort study. Arthritis Res Ther. 2021;23:1–10. 33827676 10.1186/s13075-021-02481-3 PMC8025345

[15] Trampert DC , Hubers LM , van de Graaf SFJ , Beuers U . On the role of IgG4 in inflammatory conditions: lessons for IgG4‐related disease. Biochim Biophys Acta BBA ‐ Mol Basis Dis. 2018;1864:1401–9. 10.1016/j.bbadis.2017.07.038 28782655

[16] Crescioli S , Correa I , Karagiannis P , Davies AM , Sutton BJ , Nestle FO , et al. IgG4 characteristics and functions in cancer immunity. Curr Allergy Asthma Rep. 2016;16:7. 26742760 10.1007/s11882-015-0580-7 PMC4705142

[17] Stone JH , Brito‐Zerón P , Bosch X , Ramos‐Casals M . Diagnostic approach to the complexity of IgG4‐related disease. Mayo Clin Proc. 2015;90:927–39. 26141331 10.1016/j.mayocp.2015.03.020

[18] Lighaam LC , Aalberse RC , Rispens T . IgG4‐related fibrotic diseases from an immunological perspective: regulators out of control? Int J Rheumatol. 2012;2012:789164. 22701488 10.1155/2012/789164 PMC3373157

[19] Perugino CA , Stone JH . IgG4‐related disease: an update on pathophysiology and implications for clinical care. Nat Rev Rheumatol. 2020;16:702–14. 32939060 10.1038/s41584-020-0500-7

[20] Lin W , Zhang P , Chen H , Chen Y , Yang H , Zheng W , et al. Circulating plasmablasts/plasma cells: a potential biomarker for IgG4‐related disease. Arthritis Res Ther. 2017;19:25. 28183334 10.1186/s13075-017-1231-2 PMC5301376

[21] Lanzillotta M , Della‐Torre E , Milani R , Bozzolo E , Bozzalla‐Cassione E , Rovati L , et al. Increase of circulating memory B cells after glucocorticoid‐induced remission identifies patients at risk of IgG4‐related disease relapse. Arthritis Res Ther. 2018;20:222. 30285841 10.1186/s13075-018-1718-5 PMC6235221

[22] Akiyama M , Suzuki K , Yamaoka K , Yasuoka H , Takeshita M , Kaneko Y , et al. Number of circulating follicular helper 2 T cells correlates with IgG4 and interleukin‐4 levels and plasmablast numbers in IgG4‐related disease. Arthritis Rheumatol. 2015;67:2476–81. 25989153 10.1002/art.39209

[23] Grados A , Ebbo M , Piperoglou C , Groh M , Regent A , Samson M , et al. T cell polarization toward TH2/TFH2 and TH17/TFH17 in patients with IgG4‐related disease. Front Immunol. 2017;8:235. 28348556 10.3389/fimmu.2017.00235 PMC5347096

[24] Akiyama M , Yasuoka H , Yamaoka K , Suzuki K , Kaneko Y , Kondo H , et al. Enhanced IgG4 production by follicular helper 2 T cells and the involvement of follicular helper 1 T cells in the pathogenesis of IgG4‐related disease. Arthritis Res Ther. 2016;18:167. 27411315 10.1186/s13075-016-1064-4 PMC4944254

[25] Laidlaw BJ , Lu Y , Amezquita RA , Weinstein JS , Vander Heiden JA , Gupta NT , et al. Interleukin‐10 from CD4+ follicular regulatory T cells promotes the germinal center response. Sci Immunol. 2017;2:eaan4767. 29054998 10.1126/sciimmunol.aan4767 PMC5846620

[26] Ito F , Kamekura R , Yamamoto M , Takano K , Takaki H , Yabe H , et al. IL‐10+ T follicular regulatory cells are associated with the pathogenesis of IgG4‐related disease. Immunol Lett. 2019;207:56–63. 30658078 10.1016/j.imlet.2019.01.008

[27] Perugino CA , AlSalem SB , Mattoo H , Della‐Torre E , Mahajan V , Ganesh G , et al. Identification of galectin‐3 as an autoantigen in patients with IgG4‐related disease. J Allergy Clin Immunol. 2019;143:736–745.e6. 29852256 10.1016/j.jaci.2018.05.011 PMC6265117

[28] Zhang X , Zhang P , Li J , He Y , Fei Y , Peng L , et al. Different clinical patterns of IgG4‐RD patients with and without eosinophilia. Sci Rep. 2019;9:16483. 31712579 10.1038/s41598-019-52847-6 PMC6848131

[29] Satoguina JS , Adjobimey T , Arndts K , Hoch J , Oldenburg J , Layland LE , et al. Tr1 and naturally occurring regulatory T cells induce IgG4 in B cells through GITR/GITR‐L interaction, IL‐10 and TGF‐beta. Eur J Immunol. 2008;38:3101–13. 18924213 10.1002/eji.200838193

[30] Mattoo H , Mahajan VS , Maehara T , Deshpande V , Della‐Torre E , Wallace ZS , et al. Clonal expansion of CD4(+) cytotoxic T lymphocytes in patients with IgG4‐related disease. J Allergy Clin Immunol. 2016;138:825–38. 26971690 10.1016/j.jaci.2015.12.1330 PMC5014627

[31] Akiyama M , Suzuki K , Kassai Y , Miyazaki T , Morita R , Yoshimura A , et al. Resolution of elevated circulating regulatory T cells by corticosteroids in patients with IgG4‐related dacryoadenitis and sialoadenitis. Int J Rheum Dis. 2016;19:430–2. 26385129 10.1111/1756-185X.12725

[32] Mattoo H , Stone JH , Pillai S . Clonally expanded cytotoxic CD4+ T cells and the pathogenesis of IgG4‐related disease. Autoimmunity. 2017;50:19–24. 28166682 10.1080/08916934.2017.1280029 PMC5880292

[33] Furukawa S , Moriyama M , Tanaka A , Maehara T , Tsuboi H , Iizuka M , et al. Preferential M2 macrophages contribute to fibrosis in IgG4‐related dacryoadenitis and sialoadenitis, so‐called Mikulicz's disease. Clin Immunol. 2015;156:9–18. 25450336 10.1016/j.clim.2014.10.008

[34] Della‐Torre E , Rigamonti E , Perugino C , Sain SB , Sun N , Kaneko N , et al. B lymphocytes directly contribute to tissue fibrosis in IgG4‐related disease. J Allergy Clin Immunol. 2020;145:968–981.e14. 31319101 10.1016/j.jaci.2019.07.004 PMC6960365

[35] Grados A , Vaysse T , Ebbo M , Carbonnel F , Schleinitz N . IgG4‐related disease in monozygotic twins: a case report. Ann Intern Med. 2017;166:153–5. 28114468 10.7326/L16-0122

[36] Karim F , Loeffen J , Bramer W , Westenberg L , Verdijk R , van Hagen M , et al. IgG4‐related disease: a systematic review of this unrecognized disease in pediatrics. Pediatr Rheumatol Online J. 2016;14:18. 27012661 10.1186/s12969-016-0079-3 PMC4807566

[37] de Sainte MB , Ebbo M , Grados A , Rebours V , Reumaux H , Briantais A , et al. A descriptive study of IgG4‐related disease in children and young adults. Autoimmun Rev. 2022;21:103035. 34995766 10.1016/j.autrev.2022.103035

[38] Ishikawa Y , Terao C . Genetic analysis of IgG4‐related disease. Mod Rheumatol. 2020;30:17–23. 31104539 10.1080/14397595.2019.1621000

[39] Uchida K , Masamune A , Shimosegawa T , Okazaki K . Prevalence of IgG4‐related disease in Japan based on Nationwide Survey in 2009. Int J Rheumatol. 2012;2012:358371. 22899936 10.1155/2012/358371 PMC3415093

[40] Uchida K , Tanaka T , Gershwin ME , Okazaki K . The geoepidemiology and clinical aspects of IgG4‐related disease. Semin Liver Dis. 2016;36:187–99. 27466790 10.1055/s-0036-1584323

[41] Ryu JH , Sekiguchi H , Yi ES . Pulmonary manifestations of immunoglobulin G4‐related sclerosing disease. Eur Respir J. 2012;39:180–6. 21719489 10.1183/09031936.00025211

[42] Corcoran JP , Culver EL , Anstey RM , Talwar A , Manganis CD , Cargill TN , et al. Thoracic involvement in IgG4‐related disease in a UK‐based patient cohort. Respir Med. 2017;132:117–21. 29229083 10.1016/j.rmed.2017.10.005

[43] Saraya T , Ohkuma K , Fujiwara M , Miyaoka C , Wada S , Watanabe T , et al. Clinical characterization of 52 patients with immunoglobulin G4‐related disease in a single tertiary center in Japan: special reference to lung disease in thoracic high‐resolution computed tomography. Respir Med. 2017;132:62–7. 29229107 10.1016/j.rmed.2017.09.006

[44] Muller R, Habert P, Ebbo M, Graveleau J, Groh M, Launay D, et al. Thoracic involvement and imaging patterns in IgG4‐related disease. Eur Respir Rev. 2021;30:210078. 10.1183/16000617.0078-2021 PMC9488667 34615698

[45] Ebbo M , Daniel L , Pavic M , Sève P , Hamidou M , Andres E , et al. IgG4‐related systemic disease: features and treatment response in a French cohort: results of a multicenter registry. Medicine. 2012;91:49–56. 22198501 10.1097/MD.0b013e3182433d77

[46] Chen H , Lin W , Wang Q , Wu Q , Wang L , Fei Y , et al. IgG4‐related disease in a Chinese cohort: a prospective study. Scand J Rheumatol. 2014;43:70–4. 24134471 10.3109/03009742.2013.822094

[47] Morales AT , Cignarella AG , Jabeen IS , Barkin JS , Mirsaeidi M . An update on IgG4‐related lung disease. Eur J Intern Med. 2019;66:18–24. 31227290 10.1016/j.ejim.2019.06.010

[48] Fei Y , Shi J , Lin W , Chen Y , Feng R , Wu Q , et al. Intrathoracic involvements of immunoglobulin G4‐related sclerosing disease. Medicine. 2015;94:e2150. 26683924 10.1097/MD.0000000000002150 PMC5058896

[49] Muller R , Ebbo M , Habert P , Torrents J , Gaubert JY , Schleinitz N . Pulmonary IgG4‐related disease with favourable response to rituximab: a case report. Respirol Case Rep. 2022;10:e01061. 36330374 10.1002/rcr2.1061 PMC9623431

[50] Inoue D , Zen Y , Komori T , Yoshida K , Yoneda N , Kitao A , et al. CT findings of thoracic paravertebral lesions in IgG4‐related disease. AJR Am J Roentgenol. 2019;213:W99–104. 31120784 10.2214/AJR.18.20834

[51] Fujinaga Y , Kadoya M , Kawa S , Hamano H , Ueda K , Momose M , et al. Characteristic findings in images of extra‐pancreatic lesions associated with autoimmune pancreatitis. Eur J Radiol. 2010;76:228–38. 19581062 10.1016/j.ejrad.2009.06.010

[52] Kang J , Park S , Chae EJ , Song JS , Hwang HS , Kim SJ , et al. Long‐term clinical course and outcomes of immunoglobulin G4‐related lung disease. Respir Res. 2020;21:1–9. 33076916 10.1186/s12931-020-01542-6 PMC7574178

[53] Ebbo M , Grados A , Guedj E , Gobert D , Colavolpe C , Zaidan M , et al. Usefulness of 2‐[18F]‐fluoro‐2‐deoxy‐D‐glucose‐positron emission tomography/computed tomography for staging and evaluation of treatment response in IgG4‐related disease: a retrospective multicenter study. Arthritis Care Res. 2014;66:86–96. 10.1002/acr.22058 23836437

[54] Zhang J , Chen H , Ma Y , Xiao Y , Niu N , Lin W , et al. Characterizing IgG4‐related disease with 18F‐FDG PET/CT: a prospective cohort study. Eur J Nucl Med Mol Imaging. 2014;41:1624–34. 24764034 10.1007/s00259-014-2729-3 PMC4089015

[55] Carruthers MN , Khosroshahi A , Augustin T , Deshpande V , Stone JH . The diagnostic utility of serum IgG4 concentrations in IgG4‐related disease. Ann Rheum Dis. 2015;74:14–8. 24651618 10.1136/annrheumdis-2013-204907

[56] Wallace ZS , Deshpande V , Mattoo H , Mahajan VS , Kulikova M , Pillai S , et al. IgG4‐related disease: clinical and laboratory features in one hundred twenty‐five patients. Arthritis Rheumatol. 2015;67:2466–75. 25988916 10.1002/art.39205 PMC4621270

[57] Yamada K , Yamamoto M , Saeki T , Mizushima I , Matsui S , Fujisawa Y , et al. New clues to the nature of immunoglobulin G4‐related disease: a retrospective Japanese multicenter study of baseline clinical features of 334 cases. Arthritis Res Ther. 2017;19:262. 29191210 10.1186/s13075-017-1467-x PMC5709928

[58] Qi R , Chen LYC , Park S , Irvine R , Seidman MA , Kelsall JT , et al. Utility of serum IgG4 levels in a multiethnic population. Am J Med Sci. 2018;355:61–6. 29289265 10.1016/j.amjms.2017.08.014

[59] Chen LY , Wong PC , Noda S , Collins DR , Sreenivasan GM , Coupland RC . Polyclonal hyperviscosity syndrome in IgG4‐related disease and associated conditions. Clin Case Rep. 2015;3:217–26. 25914812 10.1002/ccr3.201 PMC4405305

[60] van der Gugten G , DeMarco ML , Chen LYC , Chin A , Carruthers M , Holmes DT , et al. Resolution of spurious immunonephelometric IgG subclass measurement discrepancies by LC‐MS/MS. Clin Chem. 2018;64:735–42. 29352044 10.1373/clinchem.2017.282319

[61] Kubo S , Nakayamada S , Tanaka Y . Immunophenotype involved in IgG4‐related disease. Mod Rheumatol. 2019;29:226–30. 30334637 10.1080/14397595.2018.1537962

[62] Brito‐Zerón P , Ramos‐Casals M , Bosch X , Stone JH . The clinical spectrum of IgG4‐related disease. Autoimmun Rev. 2014;13:1203–10. 25151972 10.1016/j.autrev.2014.08.013

[63] Iaccarino L , Talarico R , Bozzalla‐Cassione E , Burmester GR , Culver EL , Doria A , et al. Blood biomarkers recommended for diagnosing and monitoring IgG4‐related disease. Considerations from the ERN ReCONNET and collaborating partners. Clin Exp Rheumatol. 2022;40(Suppl 134):71–80. 35238758 10.55563/clinexprheumatol/qq9qup

[64] Zhou J , Peng Y , Peng L , Wu D , Li J , Jiang N , et al. Serum IgE in the clinical features and disease outcomes of IgG4‐related disease: a large retrospective cohort study. Arthritis Res Ther. 2020;22:1–12. 33097076 10.1186/s13075-020-02338-1 PMC7583198

[65] Hao M , Liu M , Fan G , Yang X , Li J . Diagnostic value of serum IgG4 for IgG4‐related disease: a PRISMA‐compliant systematic review and meta‐analysis. Medicine. 2016;95:e3785. 27227950 10.1097/MD.0000000000003785 PMC4902374

[66] Campochiaro C , Ramirez GA , Bozzolo EP , Lanzillotta M , Berti A , Baldissera E , et al. IgG4‐related disease in Italy: clinical features and outcomes of a large cohort of patients. Scand J Rheumatol. 2016;45:135–45. 26398142 10.3109/03009742.2015.1055796

[67] Handa T , Matsui S , Yoshifuji H , Kodama Y , Yamamoto H , Minamoto S , et al. Serum soluble interleukin‐2 receptor as a biomarker in immunoglobulin G4‐related disease. Mod Rheumatol. 2018;28:838–44. 29251035 10.1080/14397595.2017.1416739

[68] Culver EL , Sadler R , Bateman AC , Makuch M , Cargill T , Ferry B , et al. Increases in IgE, eosinophils, and mast cells can be used in diagnosis and to predict relapse of IgG4‐related disease. Clin Gastroenterol Hepatol. 2017;15:1444–1452.e6. 28223204 10.1016/j.cgh.2017.02.007 PMC5592233

[69] Cheuk W , Chan JKC . Lymphadenopathy of IgG4‐related disease: an underdiagnosed and overdiagnosed entity. Semin Diagn Pathol. 2012;29:226–34. 23068302 10.1053/j.semdp.2012.07.001

[70] Wick MR , O'Malley DP . Lymphadenopathy associated with IgG4‐related disease: diagnosis & differential diagnosis. Semin Diagn Pathol. 2018;35:61–6. 29157939 10.1053/j.semdp.2017.11.006

[71] Liu J , Liu Y , Shen X , He Z , Yu T , Pang L , et al. Clinicopathological characteristics of IgG4‐related lung disease. BMC Pulm Med. 2021;21:1–11. 34911521 10.1186/s12890-021-01781-3 PMC8672518

[72] Zen Y , Inoue D , Kitao A , Onodera M , Abo H , Miyayama S , et al. IgG4‐related lung and pleural disease: a clinicopathologic study of 21 cases. Am J Surg Pathol. 2009;33:1886–93. 19898222 10.1097/PAS.0b013e3181bd535b

[73] Shrestha B , Sekiguchi H , Colby TV , Graziano P , Aubry M‐C , Smyrk TC , et al. Distinctive pulmonary histopathology with increased IgG4‐positive plasma cells in patients with autoimmune pancreatitis: report of 6 and 12 cases with similar histopathology. Am J Surg Pathol. 2009;33:1450–62. 19623032 10.1097/PAS.0b013e3181ac43b6

[74] Zen Y , Nakanuma Y . IgG4‐related disease: a cross‐sectional study of 114 cases. Am J Surg Pathol. 2010;34:1812–9. 21107087 10.1097/PAS.0b013e3181f7266b

[75] Takanashi S , Akiyama M , Suzuki K , Otomo K , Takeuchi T . IgG4‐related fibrosing mediastinitis diagnosed with computed tomography‐guided percutaneous needle biopsy. Medicine. 2018;97:e10935. 29851832 10.1097/MD.0000000000010935 PMC6393095

[76] Peikert T , Shrestha B , Aubry MC , Colby TV , Ryu JH , Sekiguchi H , et al. Histopathologic overlap between fibrosing mediastinitis and IgG4‐related disease. Int J Rheumatol. 2012;2012:e207056. 10.1155/2012/207056 PMC3357960 22654916

[77] Moriyama M , Ohta M , Furukawa S , Mikami Y , Tanaka A , Maehara T , et al. The diagnostic utility of labial salivary gland biopsy in IgG4‐related disease. Mod Rheumatol. 2016;26:725–9. 26873153 10.3109/14397595.2016.1148225

[78] Peng L , Zhang P , Li J , Liu Z , Lu H , Zhu L , et al. IgG4‐related aortitis/periaortitis and periarteritis: a distinct spectrum of IgG4‐related disease. Arthritis Res Ther. 2020;22:103. 32366271 10.1186/s13075-020-02197-w PMC7197178

[79] Ebbo M , Grados A , Bernit E , Vély F , Boucraut J , Harlé J‐R , et al. Pathologies associated with serum IgG4 elevation. Int J Rheumatol. 2012;2012:602809. 22966232 10.1155/2012/602809 PMC3433130

[80] Moussiegt A , Müller R , Ebbo M , Grados A , Graveleau J , Ackermann F , et al. IgG4‐related disease and hypereosinophilic syndrome: overlapping phenotypes. Autoimmun Rev. 2021;20:102889. 34237420 10.1016/j.autrev.2021.102889

[81] Santos YAP , Silva BRA , Lira PNZBA , Vaz LCA , Mafort TT , Bruno LP , et al. Eosinophilic granulomatosis with polyangiitis (formerly known as Churg‐Strauss syndrome) as a differential diagnosis of hypereosinophilic syndromes. Respir Med Case Rep. 2017;21:1–6. 28337408 10.1016/j.rmcr.2017.03.006 PMC5352719

[82] Arora K , Rivera M , Ting DT , Deshpande V . The histological diagnosis of IgG4‐related disease on small biopsies: challenges and pitfalls. Histopathology. 2019;74:688–98. 30408214 10.1111/his.13787

[83] Umehara H , Okazaki K , Kawa S , Takahashi H , Goto H , Matsui S , et al. The 2020 revised comprehensive diagnostic (RCD) criteria for IgG4‐RD. Mod Rheumatol Taylor & Francis. 2021;31:529–33. 10.1080/14397595.2020.1859710 33274670

[84] Carruthers MN , Stone JH , Deshpande V , Khosroshahi A . Development of an IgG4‐RD responder index. Int J Rheumatol. 2012;2012:259408. 22611406 10.1155/2012/259408 PMC3348627

[85] Wallace ZS , Khosroshahi A , Carruthers MD , Perugino CA , Choi H , Campochiaro C , et al. An international multispecialty validation study of the IgG4‐related disease responder index. Arthritis Care Res. 2018;70:1671–8. 10.1002/acr.23543 PMC6098740 29457382

[86] Khosroshahi A , Wallace ZS , Crowe JL , Akamizu T , Azumi A , Carruthers MN , et al. International consensus guidance statement on the management and treatment of IgG4‐related disease. Arthritis Rheumatol. 2015;67:1688–99. 25809420 10.1002/art.39132

[87] Iaccarino L , Talarico R , Scirè CA , Amoura Z , Burmester G , Doria A , et al. IgG4‐related diseases: state of the art on clinical practice guidelines. RMD Open. 2019;4:e000787. 30729031 10.1136/rmdopen-2018-000787 PMC6341179

[88] Hart PA , Kamisawa T , Brugge WR , Chung JB , Culver EL , Czakó L , et al. Long‐term outcomes of autoimmune pancreatitis: a multicentre, international analysis. Gut. 2013;62:1771–6. 23232048 10.1136/gutjnl-2012-303617 PMC3862979

[89] Masamune A , Nishimori I , Kikuta K , Tsuji I , Mizuno N , Iiyama T , et al. Randomised controlled trial of long‐term maintenance corticosteroid therapy in patients with autoimmune pancreatitis. Gut. 2017;66:487–94. 27543430 10.1136/gutjnl-2016-312049

[90] Carruthers MN , Topazian MD , Khosroshahi A , Witzig TE , Wallace ZS , Hart PA , et al. Rituximab for IgG4‐related disease: a prospective, open‐label trial. Ann Rheum Dis. 2015;74:1171–7. 25667206 10.1136/annrheumdis-2014-206605

[91] Ebbo M , Grados A , Samson M , Groh M , Loundou A , Rigolet A , et al. Long‐term efficacy and safety of rituximab in IgG4‐related disease: data from a French nationwide study of thirty‐three patients. PLoS One. 2017;12:e0183844. 28915275 10.1371/journal.pone.0183844 PMC5600376

[92] Yunyun F , Yu P , Panpan Z , Xia Z , Linyi P , Jiaxin Z , et al. Efficacy and safety of low dose mycophenolate mofetil treatment for immunoglobulin G4‐related disease: a randomized clinical trial. Rheumatol. 2019;58:52–60. 10.1093/rheumatology/key227 30124952

[93] Stone JH, Wallace ZS, Perugino CA, Fernandes AD, Patel P, Foster PA, et al. Final Results of an Open Label Phase 2 Study of a Reversible B Cell Inhibitor, Xmab®5871, in IgG4‐related disease [Abstract]. Arthritis Rheumatol. 2017;69(suppl 10). https://acrabstracts.org/abstract/final-results-of-an-open-label-phase-2-study-of-a-reversible-b-cell-inhibitor-xmab5871-in-igg4-related-disease/

[94] Ebbo M , De Sainte‐Marie B , Muller R , Piperoglou C , Grados A , Vély F , et al. Comment on article: “Dupilumab as a novel steroid‐sparing treatment for IgG4‐related disease” by Simpson et al. Ann Rheum Dis. 2020;81:e26. 31996366 10.1136/annrheumdis-2020-217010

[95] Simpson RS , Lau SKC , Lee JK . Dupilumab as a novel steroid‐sparing treatment for IgG4‐related disease. Ann rheum dis. 2020;79:549–50. 31857343 10.1136/annrheumdis-2019-216368

[96] Matza MA , Perugino CA , Harvey L , Fernandes AD , Wallace ZS , Liu H , et al. Abatacept in IgG4‐related disease: a prospective, open‐label, single‐arm, single‐centre, proof‐of‐concept study. Lancet Rheumatol. 2021;4:e105–12. 35425928 10.1016/S2665-9913(21)00359-3 PMC9004478

[97] Yamamoto M , Takahashi H , Takano K , Shimizu Y , Sakurai N , Suzuki C , et al. Efficacy of abatacept for IgG4‐related disease over 8 months. Ann Rheum Dis. 2016;75:1576–8. 27147710 10.1136/annrheumdis-2016-209368

